# Geographic and Racial Variation in Premature Mortality in the U.S.: Analyzing the Disparities

**DOI:** 10.1371/journal.pone.0032930

**Published:** 2012-04-17

**Authors:** Mark R. Cullen, Clint Cummins, Victor R. Fuchs

**Affiliations:** 1 General Medical Disciplines, Stanford University School of Medicine, Stanford, California, United States of America; 2 Departments of Economics and Health Research and Policy, Stanford University, Stanford, California, United States of America; Public Health Agency of Barcelona, Spain

## Abstract

Life expectancy at birth, estimated from United States period life tables, has been shown to vary systematically and widely by region and race. We use the same tables to estimate the probability of survival from birth to age 70 (S_70_), a measure of mortality more sensitive to disparities and more reliably calculated for small populations, to describe the variation and identify its sources in greater detail to assess the patterns of this variation. Examination of the unadjusted probability of S_70_ for each US county with a sufficient population of whites and blacks reveals large geographic differences for each race-sex group. For example, white males born in the ten percent healthiest counties have a 77 percent probability of survival to age 70, but only a 61 percent chance if born in the ten percent least healthy counties. Similar geographical disparities face white women and blacks of each sex. Moreover, within each county, large differences in S_70_ prevail *between* blacks and whites, on average 17 percentage points for men and 12 percentage points for women. In linear regressions for each race-sex group, nearly all of the geographic variation is accounted for by a common set of 22 socio-economic and environmental variables, selected for previously suspected impact on mortality; R^2^ ranges from 0.86 for white males to 0.72 for black females. Analysis of black-white survival chances within each county reveals that the same variables account for most of the race gap in S_70_ as well. When actual white male values for each explanatory variable are substituted for black in the black male prediction equation to assess the role explanatory variables play in the black-white survival difference, residual black-white differences at the county level shrink markedly to a mean of −2.4% (+/−2.4); for women the mean difference is −3.7% (+/−2.3).

## Introduction

**Figure 1:**
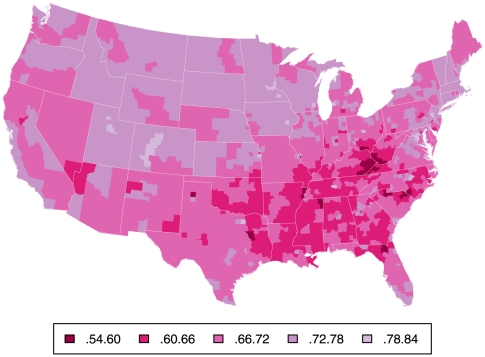
Probability of survival to age 70 for white males. Probability of S_70_ for white males by county, based on mortality rates 1999–2001. Small counties have been aggregated into Public Use Microdata Areas of >100,000 persons (N = 957).

**Figure 2:**
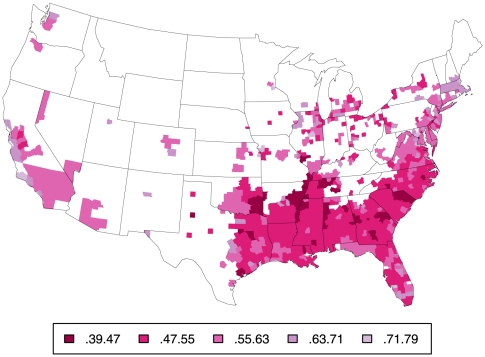
Probability of survival to age 70 for black males. Probability of S_70_ for black males by county, based on mortality rates 1999–2001. Same method as for Figure 1 for counties with sufficient black deaths, N = 510; other counties are blank. Note the different scale from 1.

**Figure 3:**
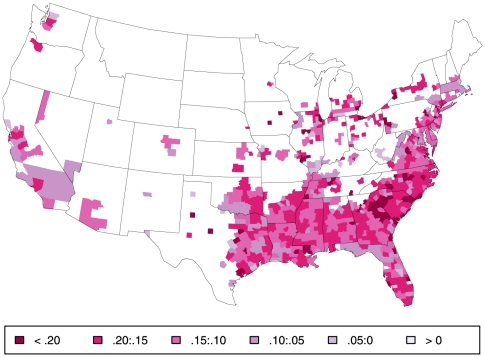
Absolute difference in survival to age 70 by county. Absolute difference in S_70_ by county between values depicted in Figure 2 (black) and Figure 1 (white). Note: Values of all differences appearing in color are negative.

Large differences in life expectancy (LE) between different regions of the country have been long recognized [1]–[2 3 4 5 6 7]. Higher mortality in large urban areas and in the South may appear at first glance attributable to regional differences in racial composition [8]–[9 10], but as illustrated by the three maps in Figures 1, 2, and 3 depicting county-level probability of survival to age 70 (S_70_) separately for white (Figure 1) and black men (Figure 2) and their difference (Figure 3), there are both salient within-race geographic differences *and* racial differences in mortality; similar gradients are seen for women (see below). Parsing evidence of this type in various ways has led some observers to conclude that there are distinct racial and geographic subpopulations living within the US, possibly with divergent and unique reasons for excess mortality [3], [ 11]–[12 13].

The sources of geographic and racial variation have been the subject of considerable research in social epidemiology, economics, demography, environmental epidemiology, behavioral sciences and health services. Employing approaches and hypotheses along largely disciplinary lines, numerous important sources of the variation have been identified and in many cases confirmed in multiple settings. Factors related to social position, including education, income and job, have been repeatedly shown to correlate strongly with mortality rates, though their *causal* importance and relative contributions have been subject to extensive debate [4], [ 14]–[15 16 17]. Region-of-origin (e.g. race-ethnicity), cultural differences (e.g., family structure), urbanization and migration-related factors have been highlighted in other studies [11], [ 18]–[19 20]. The relationships between mortality and so-called life-style choices, such as smoking, diet, and obesity have been examined from many perspectives and implicated as causes of premature mortality in cohort studies, with some evidence they may be on the pathway leading from social to regional differences [17], [ 21]–[22 23 24]. Differences in the experience of work, both as a psycho-social and possibly physical stressor, has been the focus of several studies [16], [25], [26]. Levels of ambient air pollution, most notably the small particulates generated by motor vehicles and power plants (PM_2.5_), have been implicated in differential mortality [27]–[28 29 30] as have the temperature effects based on data emerging from the climate debate [31]–[32 33]. Recent very intense investigation and reporting of regional differences in health care delivery, cost and quality [34]–[35 36 37 38], as well as evidence of historic and ongoing racial disparities in care between whites and blacks [39], [40], have highlighted the role of these factors, although estimates of their contribution to mortality rates remain uncertain.

In this report we present an ecologic model of premature mortality – death before age 70 – that includes each of the factors that could be adequately measured for both whites and blacks at the county level in order to advance understanding of the disparities in several new ways. Following Deaton, Ezzati, Murray and others [3], [8], [12] we use the whole US population as our study frame, but break the country down to the more granular county level by using as our metric of observation S_70_ rather than LE, avoiding the difficulties of estimating rates in sparse older groups and the widely observed “flattening” of race and geographic disparities observed in the study of mortality among the elderly [17], [41]. Moreover, we incorporate a broader set of predictors to bring socioeconomic, medical, environmental and demographic factors into a single model. To achieve this we employ a simplified regression analysis (weighted OLS) of county-level (ecologic) predictors of sex-specific survival to age 70 from birth separately for the white and black populations of each sex, although our aim is not so much to estimate the role of each specific factor as to describe their overall distribution and the extent to which they may collectively explain regional and race variation. This expansion of potential variables of interest is premised on the notion that racial and geographic variation most likely arises from diverse if inter-related sources. Thirdly, by demonstrating its utility to address these disparities at a granular level, we seek to establish S_70_ as an outcome measure for research beyond the better entrenched metric, life expectancy from birth.

## Methods

### Outcome measurement

**Figure 4:**
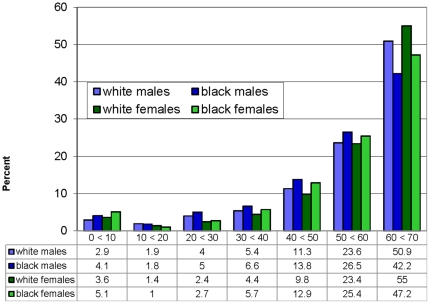
Age and distribution of deaths before age 70. The distribution of age at death for all deaths before age 70 for each subpopulation for all US in the year 2000.

We calculated the probability of survival to age 70 (S_70_) for white males, white females, black males, and black females from the CDC/NCHS Compressed Mortality Files (CMF) for the years 1999–2001 using an average of rates in the three years to reduce the effect of random or transitory circumstances that might have prevailed in 2000. Because of the change in the Census data-collection strategy, comparable more recent data are not yet available for many of the predictor variables we use (see below and Table 1). Values were obtained by applying mortality rates for each five- and ten-year interval from birth to age 70 to a child born in that county in 2000. Thus S_70_, derived like LE from period life tables, is a hypothetical statistic. It tells us what percentage of a cohort born in 2000 would survive until age 70 if the cohort experienced the age specific mortality rates that prevailed in that year. Unlike LE, which heavily “weights” events very early or late in life, S_70_, unweighted by age of death, is primarily a summary measure of mortality rates in the 40's, 50's and 60's, as illustrated in Figure 4.

**Table 1:** Predictor variables, sources, names and metrics for each county.

Construct	Variable	Variable Name	Data Source	Metric
Low educational attainment	Education<12 years	ED<12	Census	% of subgroup *10^−2^
High educational attainment	Education>12 years	ED>12	Census	% of subgroup *10^−2^
High occupational attainment	Managerial or professional job	PROF&MGR	Census	% of subgroup *10^−2^
Income	Household income per adult equivalent	INCOME	Census	Mean (Household income in$/adult equivalents) *10^−3^
Poverty	Under the poverty line	INPOV	Census	% of subgroup *10^−2^
Wealth (property)	Log of property value	PROPVALUE	Census	Mean log (property value/5×10^4^) among homeowners
Homeownership	Homeowner	HOMEOWNER	Census	% of subgroup *10^−2^
Wealth (property) distribution	Gini coefficient on property values	GINI PROP	Census	Coefficient between 0 and 1
Between race disparity in (property) wealth	Mean Black/Mean White property value	B/W INCOME	Census	Sex-specific quotient
Living without a partner	Divorced, separated or never married	SINGLE	Census	% of subgroup *10^−2^
Immigrant status	Not a US citizen	NONCITIZEN	Census	% of subgroup *10^−2^
Urban county	Metro by census definition	METRO	Census	Dummy (yes/no)
Part urban	Part metro by census definition	PARTMETRO	Census	Dummy
In the south	Southern by census definition	SOUTH	Census	Dummy
Population growth rate	Population growth rate (shrinkage) between 1990–2000	GROWTH	Census	%change ×10^−2^
Availability of fast food	Proportion of restaurant sales classified as from limited service establishments	FASTFOOD	Economic census	% sales *10^−2^
Quality of acute hospital care	Proportion of acute MI patients getting beta-blockers	BETABLOCKER	Ref	% hospitals* 10^−2^
Cold climate	Mean January temperature	JANTEMP	Ref	Degrees F*10^−2^
Warm climate	Mean July temperature	JULYTEMP	“	Degrees F*10^−2^
Air pollution	County mean concentration of fine particulate PM_2.5_	PM2.5	EPA website	PM_2.5_ in mg/M^3^
Proportion of county population that is black	Proportion of adults self-reported as black	%BLACK	Census	% *10^−2^
Black population in surrounding area	Proportion of adults in the State, excluding county, that is black	%STATEBLACK	Census	% *10^−2^

Our study design would ideally have estimated S_70_ for each sex-race group in every county, but to assure stable mortality estimates requires a minimum of 2000 total sub population in each area in the CMF. This resulted in exclusion of many hundreds of counties that had small black populations. Furthermore, the primary source for variables we used to predict S_70_ is the 5% sample of the 2000 US census, but these data are not geographically matched to the CMF. For privacy reasons the Census defines Public Use Microdata Areas (PUMAs) intended to capture 100,000+ total population areas: for low-density areas, contiguous counties were lumped together; high-density counties were sub-divided. To optimize coverage we created our own area units that match the CMF and Census geographic definitions precisely by using single counties where possible or matching groups of contiguous counties that were already grouped into PUMAs. The result is 510 areas covering 73 percent of the white and 96 percent of the black populations. They include 268 single counties and 242 groups of contiguous counties. For reader convenience, we refer to these 510 areas simply as “counties.”

### Predictor Variables

To analyze geographic differences in S_70_ we examined the relation between S_70_ in each race/sex group in each county as defined above and variation in 22 socio-economic and environmental variables that met two criteria: 1) have been broadly identified in the health literature as likely affecting mortality, hence possibly premature mortality, and 2) could be practically measured at the county level for both white and blacks (Table 1). Variables obtained from the 2000 Census of Population and Housing describe adults in each sex-race group in each county between the ages 30 to 59 with age-adjustment within that range by the direct method. Ten additional predictor variables, obtained from the Census and a wide variety of other sources, describe area characteristics; they are the same for each sex-race group except when variation in population distribution for a sex-race group affects the population weighted mean.

We would have preferred to include in our regression measures of other personal characteristics of the population which are suspect causes of premature mortality and possibly disparities, such as adverse health behaviors, diet, obesity and availability of health insurance. Although such data are sampled in periodic Behavioral Risk Factor Surveillance Surveys (BRFSS) the sample sizes for blacks are too low for all but 50 or so counties. Instead we conducted an additional sensitivity analysis (see below) to assess the importance of these covariates in explaining geographic differences among whites for whom data were adequate.

### Regressions

Multivariate (population weighted ordinary least squares) regressions of S_70_ on the 22 predictor variables were run for each sex-race group to estimate the contribution of these ecologic-level measures to geographic variation. To assess the degree to which the same 22 predictor variables explain race differences at the county level, we recalculated predicted S_70_ for black men and women after inserting the (counterfactual) corresponding *white* values for each of the predictor variables in each county, then compared the resulting hypothetical predicted value for blacks to the prediction for whites, county by county.

### Sensitivity Analysis

Because this study is limited by sample size considerations, availability of desired variables and in other ways, we carried out four complementary analyses to test the sensitivity of our results to these limitations. They are:

#### Exclusion of deaths prior to age 30

Much attention in both popular and professional publications focuses on race or sex differences in infant mortality, homicide, motor accidents, and other causes of death that are particularly important at younger ages. To determine the possible impact of omission of early life characterisitcs on our results, we repeated the analyses by examining survival to age 70 conditional on reaching age 30 (S_70/30_). Shown in Table 2.

**Table 2:** Sensitivity Analysis. Elimination of deaths under age 30 (S_70/30_), N = 510, 22 predictor variables.

	White males	White females	Black males	Black females
**Mean**	0.73	0.83	0.57	0.72
**(s.d.)**	(0.04)	(0.02)	(0.06)	(0.04)
**R^2^**	0.85	0.76	0.76	0.70

Correlation between predicted S_70/30_ and S_70_>0.99 for all four race-sex groups in 510 counties.

#### Inclusion of white counties omitted from the basic analysis

Because small black population in many counties required exclusion of many white counties, we repeated the calculation for 100 percent of the white population, which we were able to group in 957 areas of which 382 were individual counties and 575 were groups of contiguous counties. For this analysis we omitted the variable B/W INCOME for obvious reasons. Shown in Table 3.

**Table 3:** Sensitivity Analysis. Inclusion of 100 percent of whites, S_70_, N = 957, 21 predictor variables.

Mean	White males	White females	Black males	Black females
**Mean**	0.71	0.82	–	–
**(s.d.)**	(0.04)	(0.03)	–	–
**R^2^**	0.83	0.76	–	–

Correlation between predicted S_70_ for 510 counties from regressions across 957 counties and the original 510 county regression >0.99 for white males and white females.

#### Inclusion of 8 other health related variables from the Behavioral Risk Factor Surveillance Survey (BRFSS) as predictor variables

The additional variables are: current smoker, former smoker, obesity, uninsured, consumption of fruits and vegetables, physical activity, cholesterol checked, and the interaction of cholesterol check and obesity. To increase sample size, we average three years of data centered on 2000. Nevertheless, we could only make a direct comparison between the results for this augmented set of predictor variables and the results for the original 22 predictor variables for whites in 188 counties collectively comprising 51% of the US white population. Shown in Table 4.

**Table 4:** Sensitivity Analysis. Inclusion of 8 BRFSS health behavior variables, S_70_, N = 188, 29 predictor variables.

	White males	White females	Black males	Black females
**Mean**	0.72	0.82	–	–
**(s.d.)**	(0.04)	(0.02)	–	–
**R^2^**	0.90	0.86	–	–

Correlation between predicted S_70_ for 188 counties based on 29 predictor variables and predicted S_70_ for same counties based on 21 predictor variables >0.99 for white males and white females.

See Table 9 with 30 predictors.

#### Reweighting S70 based on the distribution of blacks

To assess the degree to which observed race differences might reflect differences in geographic distribution of the two races, the S_70_ for whites and blacks were weighted for each of the 510 counties by the absolute number of blacks in that county. Shown in Table 5.

**Table 5:** Sensitivity Analysis. Weighting S_70_ by black population N = 510.

	White males	White females	Black males	Black females
**Unweighted**	0.71	0.82	0.57	0.71
**Weighted**	0.69	0.81	0.54	0.70
**Difference**	0.02	0.01	0.03	0.01

## Results

### The outcome variable

Within in each sex-race group, there are striking geographical differences in the probability of survival to age 70 (S_70_) as already suggested by Figures 1 and 2. Table 6 summarizes the extent of these by comparing mean effects as well as the lowest and highest ten percent of counties within each sex-race group. These differences are larger for males than females within each race and larger for blacks than whites within each sex.

**Table 6:** Population weighted means and standard deviations of S_70_ for all 510 counties and the lowest and highest 10% of counties in each sex-race group.

	White males Mean (s.d.)	White females Mean (s.d.)	Black males Mean (s.d.)	Black females Mean (s.d.)
All 510 counties	0.71 (0.04)	0.82 (0.02)	0.54 (0.07	0.70 (0.04)
Lowest 10% of Counties	0.61 (0.02)	0.76 (0.01)	0.45 (0.03)	0.63 (0.01)
Highest 10% of Counties	0.77 (0.02)	0.85 (0.01)	0.68 (0.03)	0.77 (0.02)

**Figure 5:**
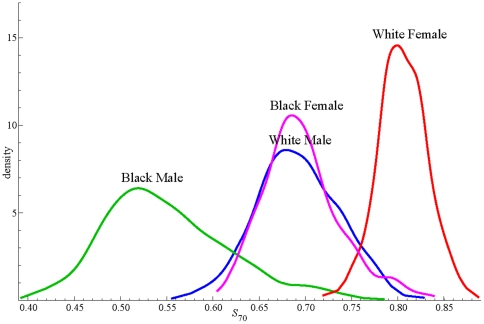
Frequency distribution (kernel plot) for S_70_. Frequency distribution (kernel plot) of survival to age 70 county for each subpopulation, 1999–2001.

For a more complete picture of inter-county differences, we show in Figure 5 the frequency distribution of S_70_ for the 510 counties for each sex-race group. The means in Table 6 have prepared us to see large differences between groups in the location of the distributions with respect to the S_70_ axis, but the fact that there is so little overlap between the distributions of blacks and whites for either sex is even more striking, as is the absence of a significant overlap of male and female distributions for either race. On average, 82 percent of a cohort of white females born today could expect to live until 70 under the assumption of unchanging mortality rates, whereas only 54 percent of black males may have that expectation. There is a significant interaction between race and sex with respect to S_70_; black-white differences are greater for males than females, and accordingly, male-female differences are greater for blacks than whites.

### Predictor Variables and Regression Results

**Figure 6:**
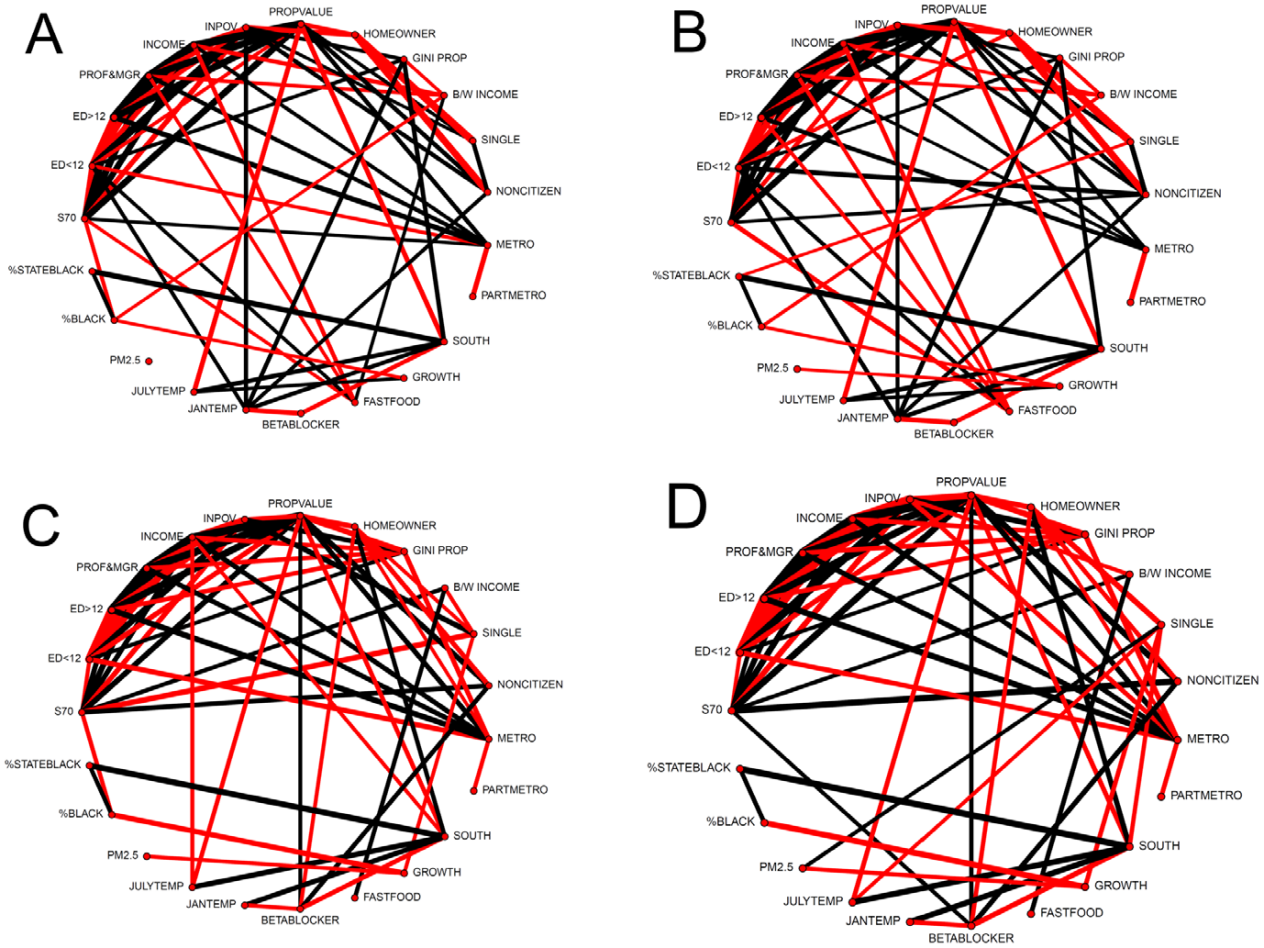
Correlation globes for the predictor and outcome variables for each of the four subpopulations, white males (A), white females (B), black males (C) and black females (D). All correlations with (absolute value) r>.36 are shown. Black lines denote a positive correlation; red negative. The thickness of the line is proportional to the absolute magnitude of the correlation.

The population weighted means and standard deviations for each of the 22 predictor variables for the four sex-race groups in each of the 510 counties are shown in Table 7. Noteworthy are the general similarities between men and women of each race, but striking between-race differences. Also noteworthy is the fact that these predictor variables are neither identically nor independently distributed. Figure 6 illustrates the highly significant inter-correlations among them for each race-sex group.

**Table 7:** Weighted means and standard deviations for each predictor variable subgroup by county, N = 510.

	White				Black			
	Males		Females		Males		Females	
	mean	std.dev	mean	std.dev	mean	std.dev	mean	std.dev
ED<12	0.13	0.05	0.11	0.05	0.25	0.08	0.20	0.07
ED>12	0.62	0.11	0.62	0.10	0.42	0.12	0.50	0.11
PROF&MGR	0.36	0.09	0.36	0.07	0.17	0.07	0.25	0.07
INCOME	0.04	0.01	0.04	0.01	0.03	0.01	0.02	0.01
INPOV	0.06	0.03	0.07	0.03	0.15	0.05	0.21	0.07
PROPVALUE	1.03	0.48	1.03	0.47	0.52	0.51	0.51	0.50
HOMEOWNER	0.77	0.10	0.78	0.09	0.58	0.12	0.54	0.12
GINI PROP	0.35	0.05	0.35	0.05	0.32	0.07	0.32	0.07
B/W INCOME	0.69	0.10	0.64	0.11	0.66	0.11	0.60	0.11
SINGLE	0.30	0.06	0.29	0.06	0.47	0.08	0.55	0.06
NONCITIZEN	0.06	0.06	0.05	0.06	0.05	0.07	0.04	0.06
METRO	0.88	0.30	0.88	0.30	0.85	0.35	0.87	0.33
PARTMETRO	0.05	0.18	0.05	0.18	0.04	0.18	0.04	0.17
SOUTH	0.39	0.49	0.39	0.49	0.55	0.50	0.54	0.50
GROWTH	0.25	0.17	0.25	0.17	0.18	0.17	0.17	0.17
FASTFOOD	0.48	0.07	0.48	0.07	0.49	0.07	0.49	0.07
BETABLOCKER	0.68	0.06	0.68	0.07	0.68	0.06	0.68	0.07
JANTEMP	0.39	0.13	0.39	0.13	0.39	0.11	0.39	0.11
JULYTEMP	0.76	0.07	0.76	0.07	0.77	0.05	0.77	0.05
PM2.5	0.14	0.03	0.14	0.03	0.14	0.03	0.15	0.03
%BLACK	0.15	0.12	0.15	0.12	0.27	0.16	0.28	0.16
%STATEBLACK	0.13	0.08	0.13	0.08	0.17	0.10	0.17	0.10

**Figure 7:**
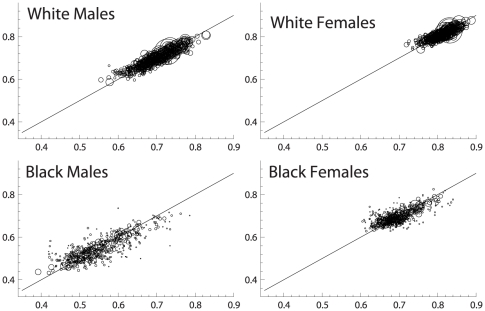
Actual S_70_ (y-axis) vs. predicted (x-axis) for each subpopulation. Note that circle size is proportional to county population (weight).

**Figure 8:**
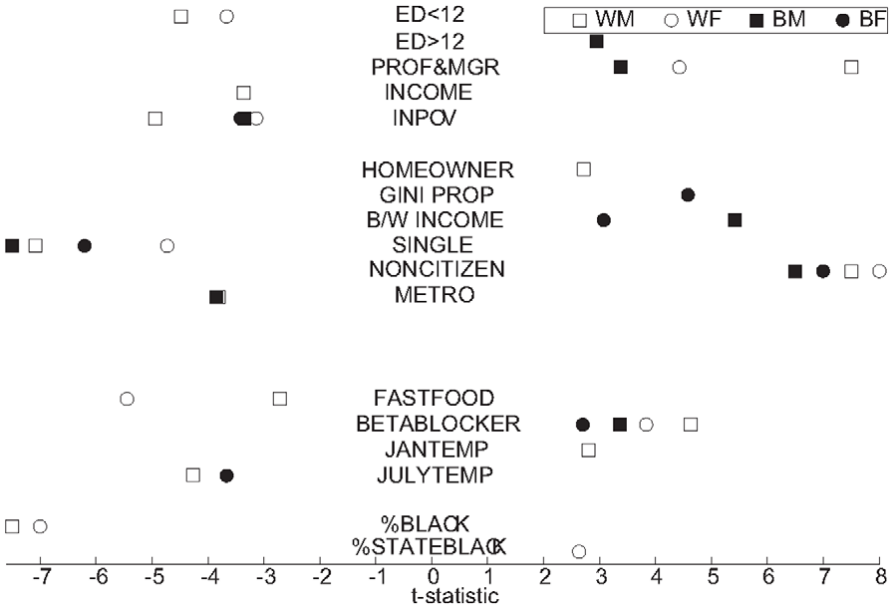
T-statistics (by sign and magnitude) for each significant predictor variable. Test statistics from the four weighted OLS regressions. Note that five variables are omitted altogether from the figure because they produced significant associations for *none* of the four subgroups: PROPVALUE, PARTMETRO, GROWTH, SOUTH and PM_2.5_.

Results of the bivariate and OLS regression of S_70_ using the 22 predictors are shown in Table 8 for each subgroup, noting the degree to which the estimated coefficients differ from the null. Notably, the percentage of variation in S_70_ accounted for by the predicted values, i.e., the regression R^2^s, are very high: 0.86 for white males, 0.79 for black males, 0.79 for white females, and 0.72 for black females; i.e., the equations account for most of the inter-county variation in S_70_ within each sex-race group. As can be seen in Figure 7, comparing the predicted and actuals for each county, the predictors are equally relevant for all levels of the distribution, and for all size counties. Figure 8 depicts the t-statistic for each individual variable for each race-sex group that falls outside the window of chance association (p<.05) in the full OLS model.

**Table 8:** Simple and multiple (weighted) regression coefficients for S_70_ for each subgroup, N = 510 counties.

	Simple regression								Multiple regression							
	White				Black				White				Black			
	Male		Female		Male		Female		Male		Female		Male		Female	
ED<12	−0.13	**	0.03		0.20	*	0.01		−0.18	**	−0.13	**	0.05		−0.09	∼
ED>12	0.22	**	0.17	**	0.42	**	0.17	**	−0.02		0.01		0.12	*	0.02	
PROF&MGR	0.32	**	0.22	**	0.60	**	0.33	**	0.24	**	0.12	**	0.21	**	0.09	∼
INCOME	2.68	**	1.72	**	6.61	**	4.44	**	−0.81	**	−0.22		−0.80		−1.52	∼
INPOV	−0.91	**	−0.36	**	−0.66	**	−0.30	**	−0.32	**	−0.13	*	−0.19	**	−0.13	**
PROPVALUE	0.06	**	0.03	**	0.07	**	0.05	**	0.01		0.00		0.01		0.01	
HOMEOWNER	0.02		−0.01		−0.08	**	−0.05	**	0.05	*	0.03	∼	−0.03		−0.02	
GINI PROP	−0.29	**	−0.08	**	−0.35	**	−0.19	**	−0.03		0.01		0.07	∼	0.10	**
B/W INCOME	−0.05	*	−0.02	∼	0.27	**	0.14	**	−0.01		0.00		0.11	**	0.04	*
SINGLE	−0.06	∼	0.05	∼	−0.50	**	−0.22	**	−0.17	**	−0.10	**	−0.28	**	−0.18	**
NONCITIZEN	0.14	**	0.15	**	0.48	**	0.40	**	0.32	**	0.32	**	0.26	**	0.31	**
METRO	0.06	**	0.03	**	0.04	**	0.03	**	−0.01	**	−0.01	∼	−0.02	**	−0.01	∼
PARTMETRO	0.01		0.00		0.01		0.01		−0.01		−0.01		0.00		0.00	
SOUTH	−0.03	**	−0.01	**	−0.03	**	−0.02	**	0.00		0.00		−0.02	∼	0.00	
GROWTH	0.03	*	0.01	∼	0.11	**	0.02	∼	0.00		0.00		0.01		0.00	
FASTFOOD	−0.23	**	−0.18	**	0.02		−0.01		−0.04	*	−0.06	**	−0.01		−0.02	
BETABLOCKER	0.18	**	0.09	**	0.31	**	0.24	**	0.07	**	0.04	**	0.11	**	0.06	*
JANTEMP	−0.05	*	0.00		0.00		−0.04	∼	0.03	*	0.02	∼	0.03		−0.03	∼
JULYTEMP	−0.21	**	−0.10	**	−0.13	∼	−0.14	**	−0.07	**	−0.02		−0.02		−0.10	**
PM2.5	−0.11		−0.11	*	−0.66	**	−0.22	*	0.01		0.01		−0.17	∼	−0.03	
%BLACK	−0.15	**	−0.07	**	−0.18	**	−0.07	**	−0.08	**	−0.05	**	−0.03	∼	0.00	
%STATEBLACK	−0.10	**	−0.03	∼	−0.13	**	−0.04	∼	0.01		0.03	*	0.05	∼	0.04	∼
Intercept									0.75	**	0.79	**	0.52	**	0.85	**
R∧2									0.86		0.79		0.79		0.72	
N	510		510		510		510		510		510		510		510	

∼ = p<.05.

* = p<.01.

** = p<.001.

**Table 9:** S_70_ weighted regressions for white males and females, 8 BRFSS variables added, N = 188 counties.

	White			
	Males		Females	
ED<12	−0.31	**	−0.24	**
ED>12	−0.02		0.01	
PROF&MGR	0.24	**	0.08	∼
INCOME	−0.78	∼	−0.19	
INPOV	−0.37	*	−0.16	∼
PROPVALUE	0.01		−0.01	
HOMEOWNER	0.03		0.01	
GINI PROP	−0.03		0.01	
B/W INCOME	−0.02		0.00	
SINGLE	−0.16	**	−0.09	*
NONCITIZEN	0.36	**	0.34	**
Current Smoker	−0.01		−0.07	**
Former Smoker	−0.03		0.02	
Obesity	0.02		0.01	
Uninsured	0.08	∼	0.03	
Fruits&Veg	0.00		0.00	
Physical Activi	0.06	*	0.00	
Chol Checked	−0.05	∼	0.00	
Chol Chk & Obes	−0.01		−0.05	
METRO	−0.02		−0.01	
PARTMETRO	−0.01		−0.01	
SOUTH	0.00		0.00	
GROWTH	0.00		0.00	
FASTFOOD	0.00		−0.06	*
BETABLOCKER	0.08	**	0.04	∼
JANTEMP	0.02		0.02	
JULYTEMP	−0.05	∼	−0.01	
PM2.5	0.07		0.03	
%BLACK	−0.08	**	−0.04	**
%STATEBLACK	0.02		0.03	∼
Intercept	0.76	**	0.86	**
R∧2	0.90		0.86	
N	188		188	

∼ = p<.05.

* = p<.01.

** = p<.001.

**Figure 9:**
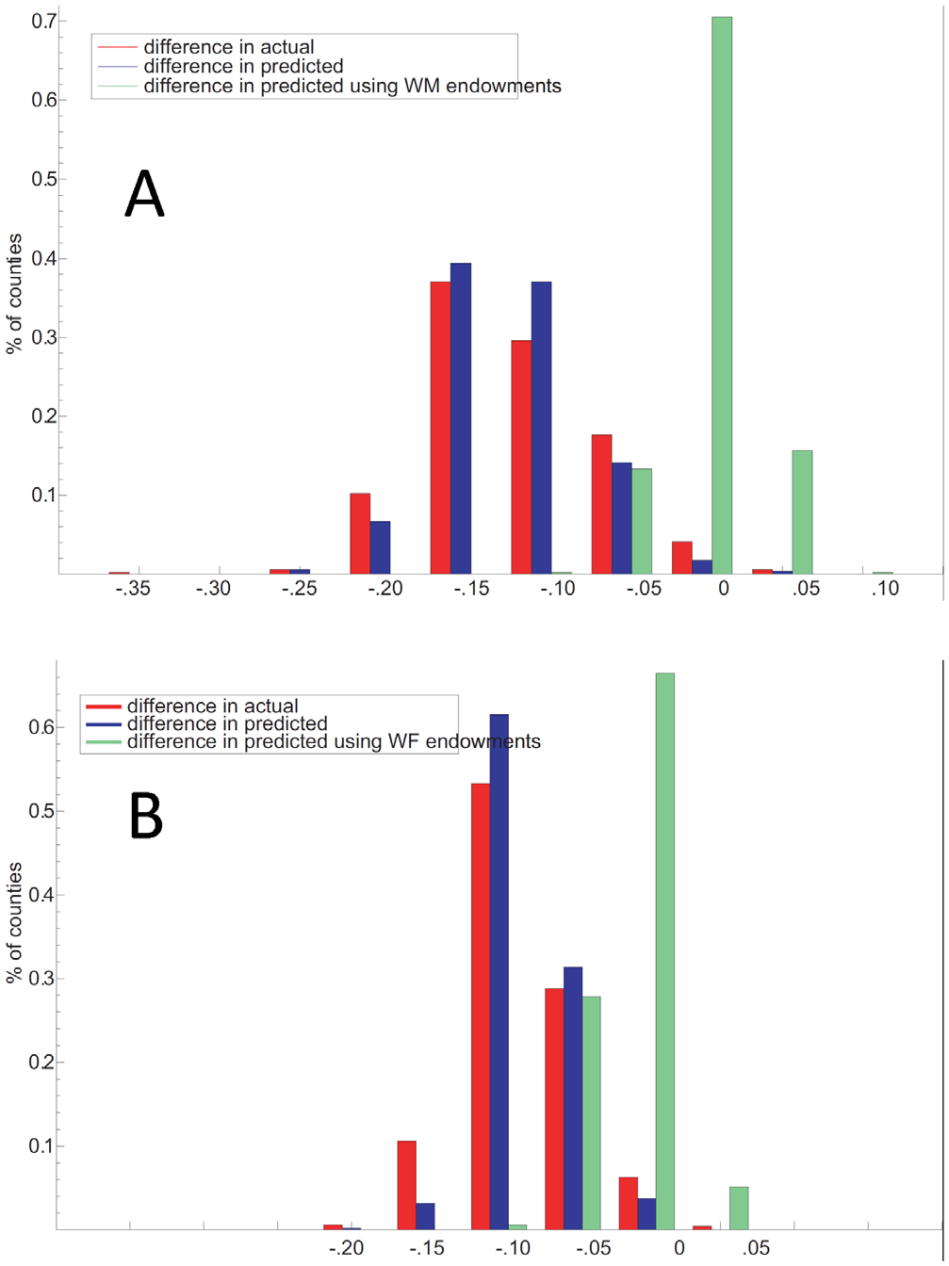
Percent of counties with actual and predicted race differences (black-white) in S_70_ for men (A) and women (B). Red and blue bars represent percent of counties (N = 510) with actual and predicted race differences (black-white) in S_70_ for men. The green bars on each panel represent the *hypothetical* black-white difference in predicted S_70_ if blacks in each county were assigned the comparable white value for each predictor variable.

We used the regression results further to examine the extent to which the race differences in distributions may be related to differences in the predictor variables. Figure 9 A and B illustrate one way to assess this. The red and blue bars on the left represent the actual (red) and predicted (blue) distributions of S_70_ for black men minus S_70_ for white men in each of the 510 counties. The green bars to the right of each panel show the results of (counterfactually) replacing the measured black values with the measured white endowments, recalculating the predicted S70 for black males under this counterfactual and hence the predicted black-white survival difference if whites and blacks were identical on the attributes. As can be seen in Figure 9A, the race differences in S_70_ at the county level narrow almost to nil: −2.4% (+/−2.4) for men, −3.7% (+/−2.3) for women (Figure 9B). When the procedure is reversed, the conclusion is the same; i.e., when black values for the predictor variables are substituted for white values in the white regressions, the curves for predicted white males (or females) resemble their black counterparts (not shown). Notably, the gender “gap” is not so explained: when female values of the 22 variables are substituted for male values in each county, there is no change in the (large) male-female differences in distribution of predicted S_70_ for both whites and blacks.

### Sensitivity Analyses

The robustness of these results was tested by four alternative approaches that varied the dependent variable, the size of the population covered, the scope of predictor variables (to include health behaviors) and the impact on our results of the different geographic distribution of whites and blacks in the US. In the first test, deaths under age 30 were excluded from the study; the dependent variable was probability of survival to 70 conditional on reaching 30 (S_70/30_). The means, standard deviations, the sex and race differences, the R^2^s, and the predicted S_70_ (r>0.99) for each county all closely match those found for S_70_.

In the second test, all the white counties that had been excluded from the basic analysis because there were insufficient blacks were included, creating a data set of 957 “counties” covering 100 percent of the white population. Again all the relevant results including predicted county S_70_ (r>0.99) closely match those obtained when 510 counties covered 73 percent of the white population.

By drawing on the BRFSS data for whites–black sample sizes were too small to allow inclusion in the main analysis–we were able to add 8 predictor variables including smoking, BMI, diet and physical activity for whites. The results, based on 188 counties covering 51% of the US white population, are very similar to those for the same counties with the 22 original predictor variables. The correlation between predicted S_70_s is very high, r>0.99. The detailed results of these sensitivity analyses are shown in Tables 2 and 3. Notably only physical activity achieves even marginal significance in a full model, and that only for men.

Finally, by weighting the S_70_ values by the number of blacks in each county, we show that geographic distribution of the races does not explain more than 1–3 percentage points of the race differential.

## Discussion

Examining the probability of survival to age 70 for each sex-race group by county we illustrate in a novel way the geographic and race disparities in premature mortality. Figure 5, with its frequency distributions of 510 counties for each of the sex-race groups, illustrates the chasmic difference between blacks and whites, true for both sexes, albeit greater for males than females. Not only are the means of these distributions significantly different as might have been expected, but there is almost no overlap: the counties with the best survival for blacks are little better than the worst counties for whites. Moreover, we have shown that differences in the 22 predictor variables, as a group, account for most of the geographic and black-white disparities in survival to age 70. Figure 7 illustrates the strength of the associations of each with S_70_ within each of the four subpopulations.

Some results, such as the impacts of education, high occupation, and marital status, are highly consistent with expectation from prior work [8], [14], [17], [41] while others—such as the failure of PM_2.5_ or prevalent behavioral factors such smoking and diet (in whites) to achieve significance—may appear surprising. We refrain, however, from drawing strong inferences about the quantitative importance of such individual observations nor do we infer from our results a causal relation between any factor and premature mortality because of limitations in our approach. First among these limitations is measurement error, which could, for example, obscure or diminish the effect of health care quality (because only a single metric was used, and that assigned fairly crudely) or PM_2.5_ that likely varies greatly within topographically diverse counties such as LA. Further misclassification of exposures are inevitable because of our treatment of time—we have used current exposure in 2000 to “predict” mortality during the same window—which may distort the role of factors with impacts over years, such as smoking and BMI. Likewise, the assumption of a linear relationship implicit in our choice of the OLS model, may be inappropriate for some variables such as income (previously shown to have a diminishing association with health [42]), while omission of other, possibly important variables, such as robust measures of health behaviors for all but the larger counties, is also a significant shortcoming in our approach. We acknowledge that the impact of change in the county composition itself, with in- and out-migration, could bias our results. We have attempted to capture such change with the single variable GROWTH but undoubtedly this is imperfect. Another factor limiting causal inference is the likelihood of reverse causality for some associations, such as health status on subsequent marital or employment status. However, with the exception of this one, most of the other limitations should tend to bias the explanatory power of our model towards the null, hence leading us to underestimate the extent to which the predictor variables as a group account for the observed geographic or black-white disparities.

Perhaps most limiting of all for causal inference is the ecologic, rather than individual level measurement of our key variables in our model because we lack knowledge of the individual characteristics of those who died. The interpretation of such models is inevitably ambiguous. For example, while we find a strong negative relation between percent in poverty and the probability of surviving to age 70, our model cannot distinguish between a) excess pre-70 deaths of individuals who are in poverty vs. b) excess pre-70 deaths of non-poor individuals who live in high poverty areas. Colinearity of some variables, as illustrated in Figure 6, may also lead to partial misattribution. Other potential limitations of this study were addressed in the sensitivity analyses with reassuring results.

While these issues collectively diminish our enthusiasm for drawing strong inferences from estimates for the individual predictors, certain observations merit comment. The very strong effects of education, poverty and occupational status across the race-sex distributions adds premature mortality to the long list of health impacts previously reported. Although the most commonly used measure of distributional disparity *within* groups (GINI PROP) showed no effect, contrary to some earlier work by [43]–[44 45 46], our result is almost identical to Deaton and others [8], [47], suggesting social disparity *between* races may be important, as suggested by the negative impact of %BLACK on white survival and the effect of black-white income differentials (B/W INCOME) on black S_70_. The impact of marital status in not a new observation [20] but the consistency of the effect across race and sex groups is noteworthy. Likewise is the very striking positive effect of NONCITIZEN—proportion immigrants is associated with higher survival in all four groups. This effect is so strong that failure to consider this variable in our model almost completely washes out the effects of education and occupation, as many of the immigrants, both black and white, have very low education attainment despite apparently better survival than their race-matched US born counterparts. This is also not a new observation, but calls further attention to likely strong health-associated selection effects first among those who come to the US who are likely to be healthier than average and later those who return to their country of origin because of poor health. This raises the possibility of statistical measurement errors for assessing mortality among such immigrant groups or possible differential impacts of the other determinants on these subpopulations [48 49]–[50].

Among the area variables, it is perhaps surprising that the classic demographic features, e.g. METRO and SOUTH, do not impart much to the aggregate association. Neither do PM_2.5_ and average temperatures, although measurement may play an important role in the failure to see such effects. FASTFOOD appears to have a measureable association for whites but not blacks, an observation that merits further evaluation. Notably, our single measure of health care quality—BETABLOCKER—shows a consistent and significant effect in all groups despite the fact it was measured at the state, not county level, likely biasing the observed effect towards the null. The impact of health care quality on survival to age 70 in the US has not, to our knowledge, been previously tested.

Taking even these observations cautiously because of the limitations, three conclusions seem inescapable. First, we have shown that geographic disparities are not primarily inherent in location, but are best understood as related to disparities in education, occupations, and the like which are strongly associated with outcome in every county we studied—large, small, urban, rural, southern or not. The absence of even a single strong outlier county (see Figure 7) lends strong support to this notion and suggest that the construct of “8 Americas” based on racial/ethnic and geographic “pockets” of poor health by Ezzati et al (3) and highlighted by others [11]–[13] is perhaps misguided. Similarly it would appear that most of the black-white gap in health is also related to differences in these well-known socio-economic and environmental variables, with poverty, low education and single marital status appearing particularly disparate between the races (cf. Table 7 and Figure 8). That this observation is not an inevitable consequence of our method is strengthened by the absence of any effect when the independent variables were “switched” between the sexes in an effort to explain the gender gap: women, perhaps due to genetic, biologic or sociologic factors omitted from our analysis, are far less susceptible than men to premature mortality attributable to their social and physical environment, at least as we have measured them.

Finally, we believe that the descriptive clarity and analytic benefits of S_70_ show it to be a useful measure of population health. While life expectancy may be useful for many purposes, such as the study of the impact of care in the elderly or changes in infant mortality to which LE is very sensitive, survival to age 70 provides an alternative measure for elucidating race and sex disparities in health. For example, while white-black difference in male life expectancy in 2006 is 7 percent, the difference in survival to age 70 is 17 percent of the average level. Female life expectancy exceeds male by 7 percent, while survival to age 70 differs by 13 percent of the average level. Not only are the differences magnified, but unlike life expectancy, S_70_ focuses unambiguously on the fact that these disparities occur for the most part in the prime, economically productive years of life (Figure 4). Alternatively we might have looked at survival to early or later ages (e.g. S_65_ or S_75_). However these choices would create other problems, at least for the US population: For S_65_ or smaller compression of the distribution at the right tail becomes a problem, as increasing numbers of counties would have values greater than 90 at least for white women. For S_75_ or greater we would likely run into many of the issues that may limit LE from birth, including flattening of the disparities at older ages and increasing relevance of late life survival factors. We suggest that overall, S_70_ may serve as the most valuable complement to that more familiar statistic used to summarize population mortality rates.
